# Hypoxia, a Targetable Culprit to Counter Pancreatic Cancer Resistance to Therapy

**DOI:** 10.3390/cancers15041235

**Published:** 2023-02-15

**Authors:** Raefa Abou Khouzam, Jean-Marie Lehn, Hemma Mayr, Pierre-Alain Clavien, Michael Bradley Wallace, Michel Ducreux, Perparim Limani, Salem Chouaib

**Affiliations:** 1 Thumbay Research Institute for Precision Medicine, Gulf Medical University, Ajman P.O. Box 4184, United Arab Emirates; 2 Institut de Science et d’Ingénierie Supramoléculaires (ISIS), Université de Strasbourg, 8 Allée Gaspard Monge, F-67000 Strasbourg, France; 3 Swiss Hepato-Pancreato-Biliary (HPB) and Transplantation Center, University Hospital Zurich, Raemistrasse 100, CH-8091 Zurich, Switzerland; 4 Department of Surgery & Transplantation, University Hospital Zurich, Raemistrasse 100, CH-8091 Zurich, Switzerland; 5 Gastroenterology, Mayo Clinic, Jacksonville, FL 32224, USA; 6 Division of Gastroenterology and Hepatology, Sheikh Shakhbout Medical City, Abu Dhabi P.O. Box 11001, United Arab Emirates; 7 Department of Cancer Medicine, Gustave Roussy Cancer Institute, F-94805 Villejuif, France; 8 INSERM UMR 1186, Integrative Tumor Immunology and Immunotherapy, Gustave Roussy, Faculty of Medicine, University Paris-Saclay, F-94805 Villejuif, France

**Keywords:** hypoxia, pancreatic cancer, immunotherapy, targeting hypoxia, vascular normalization, ITPP, hypoxia signature, hypoxia detection, genomic instability, tumor mutational burden

## Abstract

**Simple Summary:**

Hypoxia is a key feature of the tumor microenvironment involved in the pathogenesis of pancreatic ductal adenocarcinoma (PDAC). In this review we will highlight hypoxia’s integral role in shaping genomic instability and the tumor immune microenvironment in this disease. We will further present strategies currently being investigated to alleviate hypoxia and those that can be applied for its diagnosis and therapy in patients with PDAC.

**Abstract:**

Pancreatic ductal adenocarcinoma (PDAC) is the most common type of pancreatic cancer, and it is a disease of dismal prognosis. While immunotherapy has revolutionized the treatment of various solid tumors, it has achieved little success in PDAC. Hypoxia within the stroma-rich tumor microenvironment is associated with resistance to therapies and promotes angiogenesis, giving rise to a chaotic and leaky vasculature that is inefficient at shuttling oxygen and nutrients. Hypoxia and its downstream effectors have been implicated in immune resistance and could be contributing to the lack of response to immunotherapy experienced by patients with PDAC. Paradoxically, increasing evidence has shown hypoxia to augment genomic instability and mutagenesis in cancer, suggesting that hypoxic tumor cells could have increased production of neoantigens that can potentially enable their clearance by cytotoxic immune cells. Strategies aimed at relieving this condition have been on the rise, and one such approach opts for normalizing the tumor vasculature to reverse hypoxia and its downstream support of tumor pathogenesis. An important consideration for the successful implementation of such strategies in the clinic is that not all PDACs are equally hypoxic, therefore hypoxia-detection approaches should be integrated to enable optimal patient selection for achieving improved patient outcomes.

## 1. Introduction

Most pancreatic tumors (around 95%) manifest from the exocrine parenchyma of the gland, arising from connective tissue, acinar cells, or the ductal epithelium [1]. Pancreatic duct adenocarcinoma (PDAC) is the most common, accounting for more than 80% of pancreatic cancer cases [1,2]. Pancreatic cancer is associated with poor survival and the incident cases and number of deaths have reportedly doubled from 1990 to 2017 [3] and remain on a trending increase [4]. New treatment strategies are clearly necessary to enhance patient outcomes.

Immunotherapy, and in particular immune checkpoint inhibitors (ICIs), enable the activation of T cells to clear tumor cells. This is mainly by blocking inhibitory signals arising from the interaction between programmed death receptor 1 (PD1) and the cytotoxic T lymphocyte-associated protein 4 (CTLA4) with their ligands, programmed death ligand 1 (PD-L1) and B7 ligands, respectively [5]. ICIs have significantly enhanced the survival of treatment-refractory patients with metastatic melanoma [6] and non-small cell lung cancer [7], but have thus far yielded dismal responses in pancreatic cancer, even when applied in combination with chemotherapy [5,8]. The only patient subset with acceptable responses is those having microsatellite instability (MSI) or deficient mismatch repair (dMMR); in addition, high tumor mutational burden (TMB) has been significantly associated with improved overall survival after ICI treatment [9]. In patients suffering from PDAC, however, only 1–2% are mismatch repair deficient and most cases present with low TMB (<10 mutations/megabase) [10,11]. Other biomarkers of response, namely PD-L1 expression and infiltrating CD8+ T cells, have not had an established role in selecting patients with PDAC for ICI therapy [5]. An improved understanding of tumor evolution and the PDAC tumor microenvironment (TME) is necessary to bring forth more pertinent makers of responses in this disease and better combination treatments.

In this review we will briefly summarize the genetic underpinning of PDAC and the role of the TME, especially hypoxia’s, in carcinogenesis. We will further be focusing on hypoxia’s contribution to immune evasion and its impact on genomic instability. We will shed light on the concept that hypoxia could be acting as a double-edged sword, promoting tumorigenesis on one hand, and potentially increasing neoantigen production through enhanced genomic instability, on the other. We will highlight methods being applied to target hypoxia and to normalize the tumor vasculature to alleviate this condition. Finally, we will discuss approaches being used for the detection of hypoxia and how the integration of such methods for patient selection could be key for achieving the successful clinical implementation of hypoxia-alleviating strategies.

## 2. Role of Hypoxia in PDAC Carcinogenesis

Findings from various studies have been recently combined to propose an updated evolutionary model for PDAC, where often a simple *KRAS*-activating mutation transforms a ductal epithelial cell, contributing to low-grade pancreatic intraepithelial neoplasia (PanIN). Subsequent incursion of loss of tumor suppressors involved in the cell cycle control, namely *TP53*, *CDKN2A* and/or *SMAD4*, promote cell growth and the progression of the lesion to high-grade PanIN. An unknown trigger, which could be telomere loss, promotes complex mitotic errors manifesting as polyploidy in most cases, and chromothripsis in others, enables faster acquisition of structural alterations and copy number variations (CNVs). The result is rapid proliferation, heterogenous driver and pathway alterations, a compendium of transcriptional subtypes, *KRAS* allelic imbalance, invasion, and dissemination [12]. On the other hand, in *KRAS* wildtype (WT) PDACs, which represent 10.7% of cases, a recent report determined that *TP53* is the most frequently mutated gene closely followed by *BRAF* [13], while gene amplification and fusion events occur in 10% and 21% of *KRAS* WT cases, respectively [13]. In addition, a small fraction of PDACs present with single base substitution (SBS) signatures of homologous recombination deficiency (HRD) (10%) or dMMR-related signatures (1–2%). HRD and dMMR are primarily due to germline alterations in *BRCA1*, *BRCA2*, *PALB2* or *RAD51C* involved in the homologous recombination repair (HRR) pathway, or *MLH1*, *MSH2*, *MSH6* or *PMS2* involved in MMR. Germline alterations are followed by a second somatic mutation, leading to biallelic inactivation [14,15,16]. CNVs and structural variants have also been surveyed in PDAC genomes distributing them into four subtypes. This included an “unstable” subtype, which harbors greater than 200 variants [17], and was found to be associated with the HRD signature. Of interest, a recent study correlated readouts from transcriptome-based hypoxia gene signatures across tumor types with copy number signature attributes. The authors reported a significant positive correlation between hypoxia as determined by these signatures, and attributes related to HRD and aneuploidy [18]. Indeed, hypoxia has been associated with the increase in genomic instability and TMB [19,20,21,22,23,24]. In that respect, hypoxia could potentially increase neoantigen load, thus giving rise to the emergence of cancer clones that could potentially be recognized as non-self and be eliminated by the immune response. For that to be achieved however, anti-tumor immune cells need to be within the tumor mass, which is a rare scenario in PDACs that are branded as being immune-cold tumors characterized by a highly desmoplastic, hypovascularized, and hypoxic TME [25,26].

The adaptive response to hypoxia confers PDAC malignancy by promoting more aggressive and treatment-refractory phenotypes [26]. The primary activator of the hypoxia-mediated response is the hypoxia-inducible factor-1α (HIF-1α). The stability of this transcription factor is determined by the presence of oxygen since the protein responsible for initiating its degradation, prolyl hydroxylase (PHD), is activated in an oxygen-dependent manner. In the TME, the drop in oxygen levels associated with hypoxia will stabilize HIF-1α, enabling its translocation to the nucleus where it interacts with the HIF-1β subunit giving rise to a transcriptionally active heterodimer that induces the expression of more than a hundred genes. This is achieved by engaging the hypoxia response elements (HRE) in their promotor regions. Among the activated genes are those necessary for sustaining energy production in the cells, which mainly occurs via the activation of glycolysis, inhibition of oxidative phosphorylation, and by increasing the expression of glucose transporters to enable the higher uptake of this nutrient [27]. The increased intracellular acidity resulting from the hypoxia-promoted metabolic switch is counteracted though the HIF-1α induction of factors responsible for shuttling the excess lactate and hydrogen ions outside the cell. The cumulative effect is a nutrient-deprived and highly acidic TME that is hostile to the function of tumor-antagonizing cells such as cytotoxic T-cells, while being highly favorable to regulatory T cells (Tregs) and M2-polarized macrophages, which support tumor growth [28]. In addition, the leaky, haphazardly structured, and disorganized blood vessels that are instigated by hypoxia act as a physical barrier to the recruitment of immune cells [29]. A second canonical inducible activator of the response to hypoxia is the HIF-1α homologue, HIF-2α [30,31]. While there is an overlap between the responses triggered by HIFs, each isoform has a specific set of target genes [30,31]. With respect to pancreatic cancer, there have been controversial reports on the involvement of HIF-2α; however, the regulation of β-catenin by HIF-2α was found to be critical for the formation of early pancreatic lesions [32]. A more in-depth study further confirmed the crosstalk between HIF-2α and Wnt/β-catenin signaling by showing that the interaction between the two proteins increased the activity of β-catenin, while enhancing the stability of HIF-2α [33]. Furthermore, HIF-2α was shown to promote pancreatic tumor cell proliferation, metabolic shift and stemness features. It was also correlated with markers of epithelial-to-mesenchymal transition (EMT) in vivo; and with a worse prognosis in patients with pancreatic cancer [32]. In a more recent study in PDAC mouse models, HIF-2α expression in cancer-associated fibroblasts (CAFs) was found to play a key role in tumor progression and growth, as well as the recruitment of immunosuppressive immune cells to the TME [34]. Interestingly, treating PDAC mice with a HIF-2α inhibitor reduced immunosuppression and enhanced the response to immunotherapy [34]. Similar findings have been reported when combining other hypoxia-targeted approaches with ICIs, as discussed later (Section 5.3). Indeed, relieving hypoxia could transform immune-cold tumors to immune-hot and potentiate responses to immunotherapy.

## 3. Impact of Hypoxic Stress on Antigen-Specific Cell-Mediated Cytotoxicity

The benefits of cancer immunotherapy, to date, have been limited to a minority of patients. Bringing clinical benefit to the majority of patients requires a complete understanding of the mechanisms that would lead to an effective anti-tumor response and the different tumor cell-intrinsic and extrinsic factors that would result in primary, adaptive and acquired resistance to this innovative treatment approach. It has become clear that the tumor microenvironment is likely to play a crucial role in the cancer’s response to treatment. In fact, the growth and progression of cancer cells depend not only on their malignant potential, but also on the multidirectional interactions of the cellular and metabolic components of the tumor microenvironment. Most solid tumors rapidly outgrow their blood supply, leaving tumor regions with oxygen concentrations significantly lower than those found in healthy tissues. They create a hostile hypoxic microenvironment that can hamper cell-mediated immunity and dampen the efficacy of the immune response. It should be noted that in addition to a lack of oxygen and the induction of hypoxia, pseudo-hypoxic stress could take place in solid tumors. In this regard, VHL loss in clear cell renal cell carcinoma causes pseudohypoxia that profoundly alters the cellular secretome and interactions, impacting cancer renal cells and the tumor microenvironment. Clearly hypoxia plays a crucial role in tumor promotion and immune escape by controlling angiogenesis and favoring immune suppression. It is closely associated with cancer proliferation, metastasis, metabolic reprogramming, and resistance to cancer therapy. The generated hypoxic stress also has a strong impact on tumor cell biology by contributing to increasing tumor heterogeneity. It may help cells gain new functional properties and/or select certain cell subpopulations, facilitating the emergence of therapeutic-resistant cancer clones, including cancer stem cells coincident with tumor relapse and progression.

Within the adaptive immune system, cytotoxic CD8+ T cells play a central role in controlling tumor growth. We have previously investigated the mechanisms by which hypoxic stress confers tumor resistance to cell-mediated cytotoxicity including cytotoxic T lymphocytes (CTL) and natural killer (NK) cells. We have demonstrated that hypoxia-driven immune escape was multiparametric and involved different mechanisms. The inhibition of CTL-mediated lysis involves the induction of protective autophagy and stemness features in tumor cells. Furthermore, we demonstrated that the hypoxia-induced stemness transcription factor NANOG was found to be associated with autophagy activation and its binding to the BNIP3L promoter [35]. In addition, the hypoxia-induced NANOG was found to favor the intra-tumoral infiltration of regulatory T cells and macrophages via direct regulation of TGF-β1 [36]. More importantly, we found that granzyme B was degraded by autophagy, and this resulted in a decrease in tumor cell susceptibility to natural killer-mediated lysis under hypoxia [37].

Some experimental studies have revealed that the stabilization of HIF-1 by hypoxia can directly or indirectly stimulate the expression of several E-box-binding transcription factors known to regulate EMT, including TWIST1, ZEB2, and SNAIL. Using the human mammary carcinoma model MCF7, we demonstrated that MCF7 cells that experienced EMT after either introduction of SNAIL, prolonged exposure to TNF, or modulation of the WISP2–TGF–KLF4 axis, presented with resistance to CTL-mediated lysis [38]. The acquired mesenchymal phenotypes were associated with incipient stem cell-like and autophagic states, which we found to be mainly responsible for promoting reduced susceptibility to CTL-mediated lysis [39].

The inhibition of antigen-specific cell-mediated cytotoxicity by hypoxia is mediated through the impact of hypoxia on tumor target cells but also involves immune suppressive mechanisms that inhibit killer cell functions. We have shown that hypoxia induced miR-210 [40] and Tregs [36], and was able to activate myeloid-derived suppressor cells (MDSC) by inducing PD-L1 expression by these cells [41].

In addition to the effect of hypoxia on impairing the susceptibility of tumor cells to killer effectors, hypoxia plays a key role in supporting immunosuppressive cells such as regulatory T cells, MDSCs and M2 macrophages, as well as CAFs.

Hypoxia is known to be one of the central players in shaping the immune context of the TME. In this regard, hypoxia may affect the inflammatory microenvironment by modifying the polarization of macrophages, and thus reversing the inhibitory effects of a pro-inflammatory microenvironment on the malignant behaviors of cancer cells [42]. It has become clear that hypoxia shapes and induces specific macrophage phenotypes that serve tumor malignancy. Strong evidence indicates that hypoxic stress induced tumor-associated macrophages (TAM) known to mediate resistance to several anticancer treatments and to promote cancer relapse. In fact, hypoxia induces the M2-like functional transformation of TAMs by means of direct effects, orienting them to participate in immunosuppression and angiogenesis. Recently, Tregs and a number of immunosuppressive myeloid subsets, including M2 macrophages, were found to be significantly enriched in hypoxia-high tumor regions [43].

CAFs are prominent components of the microenvironment in most types of solid tumors and were shown to facilitate cancer progression by supporting extracellular matrix remodeling. We have recently demonstrated that CAFs are central players in the complex process of tumor cell–stroma interactions and are involved in the alteration of the anti-tumor immune response by impacting both cancer and immune cell populations [44].

Clearly, a deep understanding of the turbulent relationship between hypoxic stress with tumor cells in the context of stromal heterogeneity and complexity will reveal the keys to implement innovative and effective immunotherapy treatments. Based on the current knowledge of the impact of hypoxia on tumor heterogeneity, resistance and on stromal reactivity, it is tempting to speculate that targeting hypoxia within the tumor microenvironment could be one of these keys. How a smart suppression of hypoxia would be a promising strategy that is selective for facilitating immunotherapeutic efficacy in cancer patients is under investigation.

## 4. Genomic Instability in the Context of Hypoxia Influencing the PDAC Immune Response

DNA repair pathways are integral to the maintenance of genome integrity and in vitro hypoxia has been shown time and again, to depress such pathways, mainly MMR, HRR, base excision repair (BER), as well as Fanconi anemia, by down-regulating the repair genes involved. For example, severe hypoxia has been shown to down-regulate HRR effectors, including BRCA1 and RAD51, in different cancer models (reviewed in [45,46]). Furthermore, hypoxia has been implicated in promoting replication stress through the repression of DNA replication genes and stalled replication forks [19,47]. Cycling hypoxia was also shown to promote replication catastrophe in tumor cells and enhance the expression and activity of APOBEC3B (apolipoprotein B mRNA-editing catalytic polypeptide) deaminase, which is involved in tumor mutagenesis [48]. Of interest, another pertinent member of the APOBEC family, APOBEC3A, was shown to initiate chromosomal instability in pancreatic cancer, promoting metastatic progression [49].

While several studies have reported the mechanistic link between hypoxia and genomic instability in vitro, reports on the implication of this condition in pancreatic cancer have been through the analysis of patient data [21,22,23,24]. Pan-cancer genetic and molecular investigation of tumors using hypoxia signatures as a proxy to this condition led to reports that tumors classified as being more hypoxic had significantly higher genomic instability. The genomic instability was characterized by higher percentage of the genome displaying CNVs, higher load of single nucleotide variants, and structural variants [21,22]. Furthermore, hypoxia was associated with SBS signatures related to dMMR, as well as HRD [22]. Regarding tumor evolution, hypoxia was significantly associated with clonal alterations, especially structural variants, and not subclonal alterations, suggesting that the selective pressure applied by hypoxia on tumors occurs early on, and precedes subclonal diversification [22]. With respect to the subset of patients with pancreatic cancer, a high intertumoral heterogeneity was observed; nonetheless, a significantly higher genomic instability could be reported in tumors weaken higher hypoxia [21]. Of interest, when Connor and colleagues applied a different hypoxia signature to stratify 200 unpaired primary tumors and 70 metastatic tumors, as well as 11 paired primary-metastasis cases of pancreatic cancer [50], they could report no correlation between hypoxia and mutational signatures. Furthermore, no correlations were found between hypoxia and an inactivation of any specific gene by point mutations or structural variants; only a trend for biallelic loss of *TP53* could be observed at a higher frequency in the hypoxic group [50]. They also found that around half of the primary and metastatic tumors were hypoxic, and that a high concordance existed between the presence of hypoxia in paired cases, suggesting that hypoxia is an inherent feature of pancreatic tumors, and not determined solely by the TME [50]. In that respect, it would be worthwhile determining whether different levels of hypoxia could be associated with specific molecular subtypes of PDAC. Two other recent studies (discussed in Section 6.4) applied different hypoxia gene signatures to score the hypoxic state of PDAC datasets in specific and found that hypoxia was indeed associated with a higher mutation rate [23,24]. Patients classified as having more hypoxic tumors also harbored higher instances of *KRAS* mutations and *TP53* mutations [24]. In addition, in one pancreatic cancer dataset, higher MSI was present in tumors with higher hypoxia [23]. This is interesting given that the inactivation of DNA repair and mutation load have been associated with neoantigen load that could render tumor cells susceptible to elimination by the increased infiltration of immune cells [14,51,52,53].

Existing evidence of the impact of hypoxia on neoantigen load is very limited. One impactful study investigated the effect of intermittent and chronic hypoxia on genomic instability in vitro in breast cancer cells and found frameshift mutations and clonal neoantigens to be increased in both conditions [19]. These findings underline the flipside effect of hypoxia on tumor cell immunogenicity; however, whether that can be extrapolated to other tumor types, including pancreatic cancer, is unknown. While we know that pancreatic cancer is on the lower end of the spectrum with respect to TMB [54], a study on publicly available datasets of PDAC reported that almost all tissue samples presented with neoantigens that are potentially targetable [51]. It is therefore not the lack of immunogenicity that renders these tumors resistant to the immune response, but rather the presence of generally exhaustive and inactive T cells in the PDAC TME; as well as the common notion of PDACs being a desert in terms of T cell infiltration [51]. Indeed, the assessment of whole-exome sequencing data and the application of an in silico neoantigen prediction tool showed that both a high neoantigen number and an abundance of CD8+ T cell infiltrates are required to stratify such patients [55]. Furthermore, the authors showed that long-term survivors of pancreatic cancer have high-quality neoantigens that are molecular mimics of antigens derived from pathogens, which are known to evoke T cell responses. Importantly, they highlighted that it was the quality and not the quantity of neoantigens that conferred greater immunogenicity in these patients [55]. The same group recently published that neoantigen quality predicted immunoediting, wherein recurrent tumors in long-term pancreatic cancer survivors were found to have lost the clones harboring immunogenic epitopes [56]. The significance of neoantigen quality could explain the controversial findings of another study that focused only on neoantigen number and found that the lowest number was present in the patient cluster experiencing the worse overall survival [57].

The interplay among the different features of the TME and genetic underpinnings of pancreatic cancer is complex, and more work is required to better understand the implication of existing cross talks on disease pathogenicity and patient survival. Adding to the complexity is the finding that, in the great majority of pancreatic tumors, neither the number of single nucleotide variants nor neoantigen number were associated with CD8+ T cell infiltration or other markers of anti-tumor immunity. The associations only held true in cases with DNA repair deficiencies (HRD, dMMR) [58]. In that study a 4-chemokine signature was put forth and deemed necessary for eliciting a T cell-inflamed phenotype in primary and metastatic PDACs. While the authors did not investigate the implication of hypoxia on the observed phenotype, the 4-chemokine signature itself was assayed by Abou Khouzam and colleagues in PDAC patients stratified based on their hypoxic state. In that case a significantly lower expression of the signature was found in patients possessing tumors with high hypoxia compared to those with low hypoxia [23]. This patient subset was additionally more immunosuppressed (discussed in Section 6.4). Such results implicate hypoxia as an underlying trigger for signature expression and the downstream impact on the tumor’s immune context.

There is unequivocal evidence for the role hypoxia plays in tying genomic instability and immune modulation, but a lot less is known when it comes to pancreatic cancer, especially with respect to hypoxia’s implication in neoantigen quantity and quality. Given the inception of a model to predict quality neoantigens in pancreatic cancer patients, it would be of great significance to determine hypoxia’s role in this aspect of immunity. Moreover, should those neoantigens be present in hypoxic cells, using hypoxia-alleviating strategies that enable the infiltration of the tumor with immune cells could mean improved responses to ICIs.

## 5. Hypoxia-Centered Combination Therapy in PDAC

### 5.1. Targeting Tumor Hypoxia in PDAC

Pancreatic ductal adenocarcinoma (PDAC) remains the third leading cause of death related to solid cancers in men and women combined, with a poor 5-year survival rate of approximately 11% [59]. Surgery represents the only curative treatment option to date. However, due to advanced tumor stages by the time of diagnosis, less than 20% of patients qualify for surgical treatment and therefore the majority of patients undergo systemic therapy with limited effect on patient survival [60].

According to the European Society of Medical Oncology (ESMO) guidelines for the treatment of PDAC, adjuvant chemotherapy with mFOLFIRINOX (modified fluorouracil, leucovorin, irinotecan, and oxaliplatin) is the therapy of first choice in selected and fit patients. This therapeutic regimen leads to superior outcomes in terms of both disease-free survival and overall survival in R0- and R1-resected PDAC [61]. For adjuvant chemotherapy in frail patients either gemcitabine/capecitabine or gemcitabine only is recommended [1]. In cases of advanced tumor stages with metastatic disease in patients under good general conditions two types of first line chemotherapy, FOLFIRINOX or gemcitabine/nab-paclitaxel should be considered. The combination of gemcitabine/nab-paclitaxel showed a response rate of 23% and an overall survival of 8.5 months [62]. On the other hand, FOLFIRINOX showed a response rate of 31.6% and an overall survival of 11.1 months, but increased toxicity was observed [63]. Recently, data from the NAPOLI-1 trial showed that the combination of liposomal irinotecan with 5-fluorouracil and leucovorin following gemcitabine-based therapy improved overall survival, progression-free survival, disease control rate, and CA19-9 responses [64].

**Figure 1:**
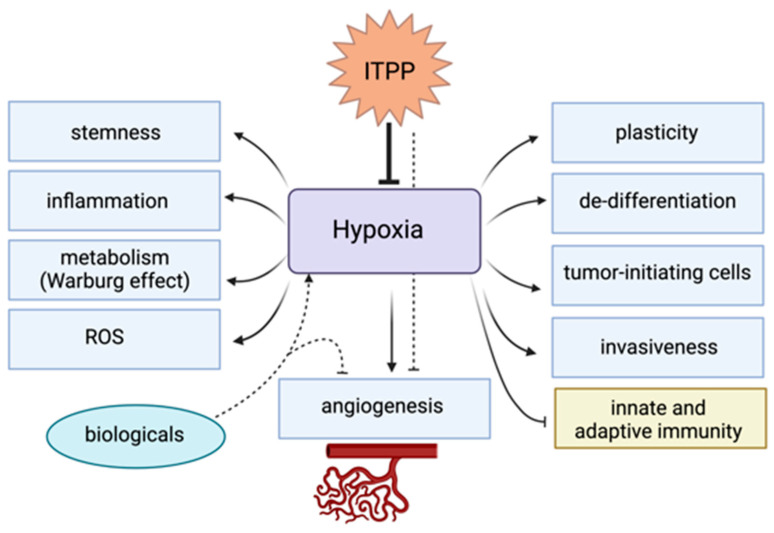
Hypoxia-mediated effects affecting various processes of tumors and the tumor microenvironment. ITPP, myo-inositol-trispyrophosphate; ROS, reactive oxygen species.

Several aspects of the poor overall survival rates in patients with PDAC are related to tumor hypoxia and hypoxia-mediated effects on tumor cells and the microenvironment (Figure 1). In their sum, these hypoxia-mediated effects lead to a more aggressive tumor phenotype, e.g., via resistance to chemotherapy, radiation therapy, and the development of early distant metastases [65,66].

Hypoxia is one of the leading causes for the aggressive behavior of PDACs and could be key in improving current therapeutic strategies [67]. Hypoxia, developing in almost all solid tumors after a specific tumor size, leads to an adaptation of tumor cells through the stabilization of hypoxia-inducible factors (HIFs) [68]. HIFs regulate hundreds of target genes involved in angiogenesis, metabolism, migration, invasion, immune escape and therapy resistance associated with tumor progression [67,69]. Moreover, hypoxia modulates various epigenetic mechanisms such as non-coding RNAs, histone modifications or DNA methylation, which interfere with HIFs [70]. So far, no therapeutic drug targeting epigenetic modifications in patients with PDAC has been evaluated. 

The levels of hypoxia vary within different tumor entities and also within the tumor itself with PDAC being exceedingly hypoxic [71,72]. One of the reasons lies in the histopathologic nature of PDAC characterized by its desmoplasia induced by pancreatic stellate cells (PSC) in addition to poor vascularization [73]. Results from a meta-analysis of eight clinical studies showed that HIF-1α overexpression correlated with a higher rate of lymph node metastasis, advanced tumor stage and was significantly associated with a poor outcome in PDACs [74]. HIF-1α contributes to stemness features by up-regulating retention in the endoplasmic reticulum which seems to be a crucial process in chemoresistance to gemcitabine [75]. Hypoxia contributes to both the direct modification of PDAC stem cell features as well as to the immunosuppressive microenvironment in PDAC tumors. Both of these pathways lead to the ineffectiveness of immunotherapy and other systemic therapies in PDACs [28,76]. Furthermore, hypoxia correlates with the production of reactive oxygen species (ROS) via the activation of PSC [77].

Several hypoxia-related antitumoral classes of medical drugs have been tested in clinical trials based on different mechanisms of action. These include counteracting hypoxia directly, targeting metabolism, HIFs, and the immune system among others (Table 1) [69]. Clinical trials investigating therapeutic hypoxia-targeting therapies in PDAC specifically are scarce and none have led to regulatory approval for market authorization so far. A randomized phase II study of PX-12, an inhibitor of the cellular redox protein thioredoxin-1 (Trx-1) located in the nucleus and cytoplasm, was performed in patients with PDAC. Increased Trx-1 gene expression correlated with HIF-1α levels in cancer cells, resulting in higher VEGF production and promoted tumor angiogenesis. This clinical trial was terminated early due to the lack of significant antitumor activity [78,79]. Moreover, compounds directly inhibiting the HIF pathway, such as PX-478, were assessed. PX-478 suppresses HIF-1α in cancer cells in preclinical rodent models and in vitro. HIF-1α inhibition combined with gemcitabine has led to reduced tumor growth and anti-tumor immunization in these preclinical studies [80] (discussed in Section 5.3, Table 2). So far, the effectiveness of HIF pathway inhibition in patients with PDAC is unknown. More recently, an early phase clinical trial assessing the direct interaction with tumor hypoxia using myo-inositol-trispyrophosphate (ITPP) has been reported [81]. The effect of acriflavine, a potent inhibitor of HIF-1α, was assessed in a preclinical trial on the growth of melanoma under normoxic conditions. The treatment with acriflavine resulted in the passing of melanoma cells due to a suppression of the microphthalmia-associated transcription factor (MITF) [82]. Another strategy of targeting hypoxic cells is via hypoxia-activated prodrugs (HAP), which are inactive prodrugs that become activated under hypoxic conditions. Moreover, several clinical trials determining the safety, tolerability, and efficacy of evofosfamide alone or in combination with other compounds are still ongoing (Table 1) A phase II trial investigating evofosfamide in combination with gemcitabine in patients with advanced pancreatic cancer showed significant improvement in progression-free survival and tumor response compared with gemcitabine treatment alone [83].

### 5.2. Antihypoxic Therapy Using Myo-Inositol-Trispyrophosphate (ITPP)

Rather than focusing on one specific aspect of tumor hypoxia, it might be more effective to target hypoxia directly (Figure 1). A new promising approach lies in the principle of re-oxygenating the tumor by anti-hypoxic therapies [84]. Inositol-tripyrophosphate (ITPP), a novel allosteric effector of hemoglobin, possesses the ability to release oxygen bound to heme under hypoxic conditions [93,94]. Preclinical studies reported that ITPP-induced restoration of tumor normoxia led to vascular normalization via down-regulation of the HIF pathway hence reducing tumor growth, invasiveness and drug resistance [95,96].

So far, ITPP has been tested in seven animal cancer models, such as pancreas, primary and secondary liver tumor, melanoma, glioma, and rhabdomyosarcoma and colon cancer models [95,96,97,98,99,100,101,102]. ITPP has been assessed in combination with systemic anticancer therapy as well as with radiation therapy. In a preclinical rodent model of PDAC, rats were treated on a weekly basis with ITPP intravenously (1.5 g/kg) for 11 weeks, while the control group received the standard cytotoxic chemotherapy based on gemcitabine via intraperitoneal injection (100 mg/kg) three times within the first week. The primary endpoint of the study was survival, tumor amount on imaging, and metastatic spread. After eight weeks, all the animals in the control group and the gemcitabine-treated group developed liver metastasis, whereas in the ITPP group metastatic spread was rarely observed in addition to restricted primary tumor growth. Furthermore, ITPP led to a significant survival benefit compared to the control group receiving chemotherapy. ITPP restored normal pO_2_ levels in tumors with a concomitant reduction in hypoxia-inducible and pro-angiogenic factors. This led to an alteration in the vascular structures from initially leaky and dysfunctional vessels to regular vascularization [97]. The same observations were additionally reported regarding counteracting chaotic and leaky tumor vasculature with the application of ITPP leading to vascular normalization in another rodent model of secondary hepatic neoplasm [95].

The first in-patient application of this novel medical drug in a phase Ib clinical study investigating the safety and tolerability of ITPP in patients with advanced gastrointestinal cancer (i.e., pancreatic ductal adenocarcinoma, colorectal cancer liver metastases, hepatocellular carcinoma, and cholangiocarcinoma) showed good tolerability and minor adverse effects. Fist insights into the efficacy of ITPP revealed radiological stabilization of disease. Moreover, when applied in a combination therapeutic regimen with subsequent systemic therapy this effect was enhanced. Additionally, a decrease in angiogenesis markers was observed in 60% of patients after the ITPP monotherapy. The authors concluded that ITPP seems to be promising when combined with chemotherapy, an effect that has to be analyzed in-depth in a phase II proof-of-concept clinical study [81].

An increasing number of ongoing clinical trials on directly and indirectly counteracting tumor hypoxia and the tumor microenvironment of PDAC shows the importance of investing this, thus far, neglected process in antitumoral drug development. Further research is urgently needed to understand the underlying cellular, epigenetic, and molecular processes responsible for PDAC remaining one of the most aggressive cancer types with limited progress in therapeutic strategies over the last decades. In addition, promising pharmacological approaches need further enhancement in order to implement findings in clinical trials.

### 5.3. Preclincal Evidence Supporting the Manipulation of Hypoxia to Enhance Responses to Immunotherapy

Hypoxia has been reported to be enriched in non-responders to immunotherapy [103]. Preclinical data on different hypoxia-targeted interventions support their combination with immunotherapy to enhance its efficacy. To date, distinct hypoxia-alleviating strategies have been applied in preclinical cancer models and were reported to revitalize the anti-tumor immune reaction and enhance responses to ICIs in different types of solid tumors [104,105,106,107,108,109,110,111,112]. Such strategies ranged from the direct targeting of hypoxic regions with hypoxia-activated prodrugs, to targeting the acidification or oxygenation and oxygen consumption within the TME, as well as targeting HIF-1α itself. In preclinical models of pancreatic cancer, hypoxia inhibitors have been applied in combination with chemotherapeutic agents or radiation therapy and have shown enhancements of treatment efficacy in terms of a reduction in tumor growth [80,113,114,115,116,117,118,119] and even improved survival [116,120] (Table 2). Importantly, immune modulation was also investigated in a fraction of these studies and combination therapy resulted in a remodeling of the immune microenvironment [80,116,120]. With respect to ICIs, only two studies have thus far tried them in combination (Table 2), one in combination with a HIF-2α inhibitor [34], and the other with oxygen microcapsules [108]. Both studies used the same PDAC mouse models and showed enhanced efficacy of ICIs when combined with hypoxia-manipulating agents. In addition, Wu J. and colleagues [108] showed increased M1 polarization and infiltration of leukocytes, with no change however in MDSC infiltrates. Therefore, preclinical data and the promising results from a phase I clinical trials testing the safety and efficacy of combined anti-CTLA4 (ipilimumab) with evofosfamide (NCT03098160, Table 1) [88], support the integration of such a multimodal approach in the clinic. More work is required to determine the most ideal hypoxia-alleviating strategy to be combined with ICIs in patients with PDAC and determine the patient subset that could benefit most from such a combination.

**Table 2:** Preclinical studies combining hypoxia-targeting strategies with conventional therapy in PDAC.

Target	Drug ^a^	Mouse Model ^b^	Combination Therapy	Immune Modulation	Efficacy of Combination	Ref.
HIF-1α	PX-478	Panc-1, CF-PAC-1 or SU.86.86 subcutaneously implanted in the flank of female SCID mice	Fractionated radiation therapy, with or without the combined treatment with 5-fluorouracil or gemcitabine	NA	Significant increase in tumor regression, potentiating the antitumor activity of radiation and chemotherapy	[113]
HIF-1α	PX-478	Panc02 subcutaneously implanted in the flank of C57BL/6 mice and immuno-incompetent nude (Nu/Nu) mice; Subcutaneous inoculation in the flank of Panc02 dying and dead cells and the supernatant from cells untreated or treated with Gem and/or PX-478 (vaccination) followed by subcutaneous implantation in the flank of surviving cells (challenge)	Gemcitabine	Increased cytotoxic CD3+/CD8+ T lymphocytes in the spleen and tumor tissues in mice compared to the single treatment	Significant reduction in tumor growth in immune-competent and incompetent mice with the single treatment; Increased tumor suppression effect in immune-competent but not in nude mice compared with the Gem single treatment; high vaccine efficacy, decreasing tumor growth by inducing immunogenic cell death	[80]
HIF-1α downstream signaling (LOX)	LOX-blocking antibody	PDAC-bearing KPC mice	Gemcitabine	Increased leukocyte, macrophage, and neutrophil infiltration compared to the single treatment	Increased survival and decreased metastatic burden	[120]
Microenvironmental hypoxia	TH-302	Patient-derived pancreatic xenografts subcutaneously implanted in the leg	Fractionated radiation therapy	NA	Decreased tumor growth in fast-growing tumors only	[114]
Microenvironmental hypoxia	TH-302	AsPC1 cells orthotopically implanted in the pancreas of (Nu/Nu) mice	Single-dose radiation therapy	NA	Significantly more effective in delaying tumor growth than the single therapy	[115]
HIF-1α downstream signaling (CA9)	SLC-0111	PK-8 or PK-1 cells subcutaneously implanted on the back of female NOD/SCID or NSG mice; Patient-derived pancreatic xenografts—subcutaneous implantation of tissue fragments into male C.B-17 SCID mice; PDAC-bearing KPCY mice	Gemcitabine	Decreased B220+ B cells with no impact on the number of CD3+ T cells in the combination treatment compared to single treatment	Decreased tumor growth and increased survival	[116]
HIF-1α	VHH212 nanobody	PANC-1 cells subcutaneously implanted in the flank of BALB/c nude mice	Gemcitabine	NA	Higher inhibition of tumor growth compared with gemcitabine alone	[117]
Microenvironmental hypoxia	Liposomal vinblastine-N-Oxide (CPD100Li)	PANC-1 cells subcutaneously implanted in the tail of female Nu/Nu mice	Gemcitabine	NA	Significant decrease in tumor growth compared to gemcitabine alone	[118]
Microenvironmental acidosis and hypoxia	Gold nanorods (GNRs)	KPC tumor cells subcutaneously implanted in male C57BL/6 mice; Luciferase-transfected KPC tumor cells orthotopically implanted in the pancreas of male C57BL/6 mice	Single-dose radiation therapy	NA	Significantly more effective in delaying tumor growth and decreasing tumor volume than the single therapy	[119]
HIF-2α	PT2399	KPC cells subcutaneously implanted into the flank of syngeneic C57BL/6 female mice; KPC cells were orthotopically implanted into the tail of the pancreas of syngeneic C57BL/6 male mice	ICI	NA ^c^	Combination treatment with anti-CTLA4 led to a significant decrease in tumor growth in the tested subcutaneous model compared to each drug alone; dual checkpoint blockade (anti-PD1 and anti-CTLA4) combination with PT2399 led to decreased tumor growth and enhanced survival in the orthotopic model (combined with anti-pd1)	[34]
Microenvironmental hypoxia	Oxygen microcapsules	KPC cells subcutaneously implanted into the flank of syngeneic C57BL/6 female mice; KPC cells were orthotopically implanted into the tail of the pancreas of syngeneic C57BL/6 male mice	ICI	Increased the infiltration of CD45+ immune cells and increased the proportion of M1 macrophages with no effect on MDSC infiltration compared to the single treatment	Combination treatment with anti-PD1 led to significant decreases in tumor growth compared to each drug alone	[108]

^a^ TH-302: also known as evofosfamide; VHH212-encoding adenovirus targeting intracellular HIF-1α; gold nanorods with a charge-reversal nanocarrier (poly(l-glutamic acid-co-l-lysine) [P(Glu-co-Lys)]) that is triggered by extracellular acidification of the tumor. ^b^ SCID mice: immune-compromised mice lacking mature B and T lymphocytes; Nu/Nu mice: athymic mice lacking T cells; KPC mice: genetically engineered mouse model for PDAC; NOD/SCID mice: immune-deficient mice with a diabetes-susceptible non-obese diabetic (NOD) background; NSG mice: NOD SCID gamma, severely immune-compromised mice; KPCY mice: genetically engineered mouse model of pancreatic cancer with pancreas-specific knockout of Yap. ^c^ Effect of HIF-2-deleted fibroblasts on immune cell population in PDAC was conducted separately. The effect of the drug on immune reactivity was only assessed in vitro and not in established tumors.

## 6. Detection of Hypoxia in the TME of Patients with PDAC

### 6.1. Direct Oxygen Quantification

The initial report on pancreatic tumors being extremely hypoxic came from the direct measurement of the oxygen partial pressure (pO_2_) of pancreatic tumors and the corresponding normal pancreatic tissues of seven patients [121]. Multiple measurements were taken per patient and the intra-tumoral median pO_2_ levels were reported to be between 0 and 5.3 mmHg, while those of the normal pancreas ranged from 9.3 to 92.7 mmHg [121]. When the grand median of pO_2_ in pancreatic cancer patients was compared to that of other tumor types, measured with the same technique, pancreatic tumors appeared to be the most hypoxic [122]. The technique of inserting micro-electrodes into tissue to measure the current generated by the reduction in oxygen at the cathode extremity has been the gold standard for measuring absolute oxygen histograms with high precision (down to 1 mmHg) [122]. The Eppendorf^®^ pO_2_ histography system, which is no longer commercially available, was equipped with a computerized drive that moved the electrode through the tissue, thus minimizing oxygen consumption by the electrode and tissue compression [123]. While hypoxia, as determined by oxygen tension measurements in this histography system, could be negatively associated with prognosis in different tumor types, this was limited to shallow tumors [122]. However, the highly invasive nature of this system, as well as the need for experienced professionals that can limit measurements from anoxic non-viable cells and non-tumor tissues, eventually stunted its clinical application [122,123]. Other techniques have been developed since then using, for example, fiber-optic devices, such as OxyLite, in which instead of consuming oxygen, the tip of the device is equipped with a fluorophore that becomes stimulated by photodiodes. The oxygen tension at the probe tip is inversely proportional to the fluorescence lifetime [124]. Such a tool though has not been applied in patients with pancreatic cancer, and the probes themselves have not attained regulatory approval for the application in human subjects [123]. Similarly, in another method for the quantitative assessment of tumor oxygenation, electron paramagnetic resonance (EPR) oximetry [125], which makes use of the paramagnetic nature of oxygen and applies oxygen reporters, has been limited in human application to superficial tumors. This again negates the potential of applying such a strategy in pancreatic cancer.

Indeed, to date, most of the techniques that have been integrated for the estimation of hypoxia in pancreatic cancer have been based on indirect measures, including immunohistochemical staining of hypoxia-related markers, imaging parameters, and gene expression signatures.

### 6.2. Immunohistochemistry (IHC)-Based Detection of Hypoxia-Related Markers

Multiple studies have assessed the endogenous hypoxia marker HIF-1α and showed it to be associated with a worse patient prognosis [74,126]. One study took a different approach by applying the exogenous tracer of hypoxia, pimonidazole. This 2-nitroimidazole is administered to patients pre-operatively and undergoes bio-reductive metabolism under low-oxygen conditions, forming stable adducts that can subsequently be detected by immunohistochemistry (IHC) in tissue sections [127]. In brief, patients received one dose of pimonidazole over a period of at least 30 min, 16 to 24 h before surgery. Resected tumors were paraffin embedded and serial sections were stained for pimonidazole using monoclonal IgG1 antibody, hypoxyprobe MAb1. Stained sections were digitized, and analysis was performed using Genie, Aperio’s pattern recognition software. This software differentiates between epithelial, stromal, and other (non-tumor) regions. Regions with positive immunostaining for pimonidazole were defined as hypoxic tumor areas. Quantification of the hypoxic percentages of the whole tumor (excluding other regions), as well as those in epithelial and stromal compartments were quantified by the Aperio’s Positive Pixel v9 algorithm. In addition, manually scoring was independently conducted by assigning the epithelial tumor compartment of each section a score representing the hypoxia percentage [127]. This study which was conducted as a prospective trial (PIMO-PANC—NCT01248637) shed light on the high inter- and intra-tumoral heterogeneity of hypoxia in PDAC. The analysis of multiple sections sampled from the same tumor showed substantial intra-tumoral heterogeneity suggesting the need for multiple sections and/or biopsy samples to be analyzed before concluding the hypoxic state. Of interest, variation in hypoxia was even more palpable between patients; such variance in hypoxia supports the feasibility of stratifying patients based on hypoxia, given adequate tumor sampling [127]. The same group recently compared the performance of three different digital imaging platforms for assessing the pimonidazole staining from ten patients in the accrued cohort [128]. They put forth a workflow for a quantitative, automated and high-throughput digital image analysis and applied it to the full PIMO PANC cohort to investigate the association of hypoxia with patient prognosis [128]; however, the results from this analysis are yet to be published.

The issues associated with robustness, broad applicability, subjectivity, and inter-observer variability in IHC analysis remain as relevant setbacks. These pitfalls could be surpassed by the integration of digital imaging platforms with trained computational algorithms that can relay quantitative results without human bias [128].

### 6.3. Imaging-Based Parameters as Hypoxia Biomarkers

The application of magnetic resonance imaging (MRI)-based parameters to interrogate diverse facets of hypoxia has been extensively tested in preclinical models and in cancer patients [129]. The dissolved dioxygen in solution with two unpaired electrons and the deoxyhemoglobin (dHb) monomer with four unpaired electrons act as endogenous contrast agents, along with water motion and relaxation times, and contribute to the parametric outputs of MRI. The relaxation times are time constants reflecting the return of the magnetization to its initial values following radiofrequency pulse excitation, and these include T1, T2 and T2* [123]. The inverse of these relaxation times are known as the relation rates R1, R2 and R2*. R1 and R2* are oxygen sensitive and potential biomarkers of tumor hypoxia [123]. These parameters however cannot be used for direct oxygen measurements since they are additionally affected by other factors [123,129]. Indeed, the correlation of R2* with other markers of hypoxia, such as pimonidazole staining or HIF-1α expression has shown controversial results [123].

Dynamic contrast-enhanced MRI (DCE-MRI) has additionally been employed following the injection of exogenous contrast agents for arterial spin labeling [129]. Among the parameters produced by DCE-MRI, Ktrans is the transfer constant of the contrast agent from plasma to the extracellular, extravascular space and is affected by blood flow, surface area of capillaries and their permeability; ve is the volume of extracellular, extravascular space and represents the leakage volume; vp is the fractional plasma volume, or the perfusion fraction; and the rate constant kep is the ratio of Ktrans to ve and indicates the transfer of contrast agents from the extracellular, extravascular space to the plasma [130,131,132]. DCE-MRI is a marker for perfusion and not an indicator of oxygen consumption and has therefore been suggested as a predictive method for the response to strategies aimed at increasing oxygen delivery by modulating blood perfusion [123,133]. With respect to pancreatic cancer, DCE-MRI has been applied in a prospective clinical trial on 11 patients with locally invasive pancreatic cancer prior to and 28-day post-combination treatment with chemotherapy and antiangiogenic therapy [130]. In this case, pre-treatment values of Ktrans could predict responses to antiangiogenic therapy and perfusion parameters were all found to decrease following combination therapy. A more recent study on 15 patients with PDAC assessed the repeatability and interaction of DCE-MRI parameters Ktrans and kep, vp and ve as well the T2* MRI parameter R2*, in the hope of putting forth a set of parameters that could cumulatively capture perfusion and hypoxia [131]. They demonstrated good repeatability in measures obtained from T2* MRI and DCE-MRI and showed that Ktrans and ve were positively correlated with T2*. They also found that tissue R2* increased with a lower tissue Ktrans, which suggests that a low Ktrans could correspond to tissue regions of lower oxygenation. However, there was no comparison with tissue markers of hypoxia, or PET radiotracers, to determine whether this parameter could be used as a surrogate for hypoxia in pancreatic cancer. The same group then applied a combinational approach to characterize tumor hypoxia, as well as vessel density and collagen fraction by matching DCE-, intravoxel incoherent motion (IVIM)-, and R2*-derived MRI parameters with tissue-based characteristics [132]. They conducted a preliminary study with MRI data on 30 treatment-naïve patients with PDAC and matched whole-mount histology in 15 patients. They found significant correlations between functional MRI parameters and histological markers of collagen (Picrosirius red (PSR) staining), vessel density (von Willebrand factor (vWF) staining) and hypoxia (HIF-1α nuclear staining). Based on histological and survival findings, they put forth two main phenotypes defined as stroma-high, exhibiting a high vessel density and a collagen fraction, and stroma-low, having a low vessel density and a collagen fraction. It could be expected that patients in the stroma-low group are prone to having more hypoxic tumors due to the presence of a lower vessel density. Indeed, those patients experienced significantly worse OS and DFS than patients in the stroma-high group [132]. However, while an association could be found between R2* and hypoxia, as indicated by HIF-1α nuclear staining, vascularization and diffusivity could not be correlated to HIF-1α positivity.

In the scope of imaging-based tools, positron emission tomography (PET) scanning coupled with a radiotracer has been heavily explored in different tumor types, including pancreatic cancer. The overarching principle of this method entails a radiotracer being injected into the patient hours before the scan. The radiotracer will cross the membranes of normoxic and hypoxic cells. However, it will only be reduced in hypoxic cells, forming adducts with macromolecules withing these cells. Normoxic cells will eventually clear the radiotracer. Three main types of such tracers exist, all having a radiolabeled fluorine, ^18^F, ^18^F-FMISO (fluoromisonidazole), ^18^F-FAZA (fluoroazomycin–arabinofuranoside) and ^18^F-HX4 (flortanidazole) [134,135,136,137]. As can be seen in Table 3, each study that applied such tracers in pancreatic cancer, computed a different parameter to represent hypoxia and they were all limited in their sample size, making it difficult to extend the proposed methods beyond the tested cohorts. Recently, the feasibility of applying ^18^F-FAZA to guide adaptive radiation dose-escalation based on hypoxic volumes was simulated in patients with locally advanced, unresectable pancreatic cancer, where a model was derived that converts FAZA PET images of pancreatic tumors to oxygen-enhancement ratio-maps. Through their model, the authors found that in comparison with standard radiotherapy, stereotactic body radiation therapy (SBRT) with dose painting of hypoxic volumes resulted in a bigger reduction in clonogenic cell survival fractions [138].

While imaging-based techniques could enable the longitudinal assessment of hypoxia and its visualization in the context of the entire tumor mass, the primary issue with these methods is their standardization, which would be critical for their successful implementation on a large scale.

### 6.4. Gene Signatures as Hypoxia Surrogates

Hypoxia and the stabilization of HIF1 modulates several pathways ensuring the survival of tumor cells through immune evasion, imparting EMT and stemness features, genomic instability, and metabolic reprogramming (Figure 1). The expression of genes involved in such downstream pathways have been integrated in diverse studies to develop hypoxia gene signatures with prognostic relevance in solid tumors. While different approaches have been used to derive and test the association of gene signatures with patient outcomes, the unanimous result has been that hypoxia, as determined by these signatures, is associated with worse survival (reviewed in [29,139,140]).

With respect to pancreatic cancer, a study that included a cohort of 73 patients, found hypoxia signatures to be enriched in those experiencing worse survival [120]. In another study, 76 validated hypoxia genes were distilled down to thirty genes, and the median expression of these genes was used to score the degree of hypoxia in pancreatic patients [50]. OS and progression-free survival (PFS) analysis were then carried out on 148 primaries. Hypoxia was reported as one of the tumor features associated with worse OS and PFS following univariate survival analysis with Cox proportional hazard. However, the association was lost in multivariate analysis. The authors also showed in a small cohort of 11 patients who received adjuvant therapy prior to resection, that hypoxia was absent in patients with partial response and more enriched in those with stable disease (six out of seven), suggesting its predictive power of response to therapy [50]. Since that report, six studies to date have each associated distinct hypoxia gene signatures with worse overall survival in pancreatic cancer patients [23,24,141,142,143,144] (Table 4). The integration of different derivation methods and datasets or cancer cell lines to put forth a hypoxia signature, meant that each study reported a different set of genes (Table 4). Of interest, four genes were present in more than one signature, namely *LDHA* (four signatures), *PGK1* (two signatures), *ENO3* (two signatures) and *TES* (two signatures). *LDHA* and *PGK1* are validated hypoxia genes, with known HRE elements and key roles in metabolic reprogramming. *ENO3* is a gene involved in the glycolytic pathway, but seemingly contributes to improved prognosis, with lower levels associated with worse survival in pancreatic cancer [142,143,145]. With respect to *TES*, while it has been reported to act as a tumor suppressor in other tumor types [146,147], in pancreatic cancer its increased expression seems to be associated with worse survival [143,144].

Stratification of patients has been conducted either based on a hypoxia score that was calculated considering median gene expression [23,141] or according to a hypoxia risk score, which was derived by including regression analysis to determine the contribution power of each gene to survival and using that factor to calculate a risk score [24,142,143,144] (Table 4). One advantage of patient stratification is that other aspects of the tumor microenvironment can be explored, including the immune context and molecular features. Indeed, most of the studies reporting on pancreatic cancer hypoxia gene signatures conducted additional exploratory analysis of the immune context, by subjecting the transcriptomes of patients in each group to immune-cell fraction analysis tools, such as CIBERSORT (cell-type identification by estimating relative subsets of RNA transcripts), or gene set enrichment analysis, or even direct exploration of differential expression of immune-related genes. Results of such analysis clearly point to hypoxia’s association with an immunosuppressive TME in pancreatic cancer (Table 4).

**Table 4:** Prognostic hypoxia gene signatures in pancreatic cancer.

	Signature and Cohort Characteristics				Survival Analysis ^a^				Immune Analysis ^b^			
Genes	Derivation	Scoring	Cohort	Groups (Patient Number) ^c^	End Point	KM (*p*-Value)	Univariate Cox PH	Multivariate Cox PH	Method	High-Risk Group (Hypoxia-High)	Low-Risk Group (Hypoxia-Low)	Ref.
30 ^d^	Overlap between 200 genes of the hallmark HYPOXIA gene-set and microarray data of two pancreatic cancer cohorts (GSE15471 and GSE16515)—30 DEGs	Gene score: +1 if gene expression > median expression in entire cohort; −1 if < median expression in entire cohort. Hypoxia score is sum of 30 genes	PAAD TCGA	High (79) vs. Low (98)	OS PFS	0.0062 0.0024	NA	NA	NA	NA	NA	[141]
8 (*DDIT4*, *LDHA*, *MXI1*, *NDRG1*, *P4HA1*, *PGK1*, *SLC2A1*, *VEGFA*)	Expression of 15 genes selected from 398 hypoxia genes collected from published prognostic or predictive signatures tested in 14 cancer cell lines exposed to 1% oxygen	Gene score: +1 if gene expression > median expression in entire cohort; −1 if <median expression in entire cohort. Hypoxia score is sum of 8 genes	PAAD TCGA	High (66) vs. Low (98)	OS DSS PFS	0.0035 0.0047 0.01	1.9 (1.2–2.9) *p* = 0.004 2 (1.2–3.2) *p* = 0.005 1.7 (1.1–2.5) *p* = 0.011	1.7 (1.10–2.7) *p* = 0.016 1.6 (0.99–2.6) *p* = 0.056 1.5 (0.97–2.2) *p* = 0.067	CIBERSORTx Immune score Cytolytic index 4-chemokine signature	M0 macrophages, low cytolytic index, low immune score and low chemokine score	CD8+ T cells, high cytolytic index, high immune score and high chemokine score	[23]
			E-MTAB-6134	High (136) vs. Low (173)	OS DFS	<0.0001 <0.0001	2.1 (1.6–2.8) *p* < 0.001 1.8 (1.3–2.3) *p* < 0.001	2.19 (1.60–3.0) *p* < 0.001 1.8 (1.39–2.5) *p* < 0.001				
9 (*ARNTL1*, *DCBLD2*, *DSG3*, *FAM83A*, *FOXM1*, *GZMK, IGF2BP2*, *SLC38A11*, *TPX2)*	15 overexpressed HIF-1 related genes in meta-PDAC cohort (GSE62452 and PAAD TCGA)—nine showed critical prognosis association using LASSO regression analysis	Multiplying expression of nine genes with their corresponding multivariable Cox regression coefficient—classification into high-, medium- and low-score based on cutoffs determined by X-tile 3.6.1 software	Meta-PDAC cohort	High (22) vs. Medium (73) vs. Low (110)	OS	5.584 × 10^−14^	2.276 (1.741–2.975) *p* < 0.001	2.162 (1.632–2.865) *p* < 0.001	Enrichment scores of 25 immune-related terms determined from previous studies in the meta-PDAC cohort only immunostaining for CD8+ T cells in 28 PDACs sorted into low- and high-HIF-1 scores based on median cutoff of HIF-1 scores determined using RT-qPCR	TIL, activated CD8+ T cells, cytolytic activity, activated B cell, immature B cell and Type 1 T-helper cells significantly more enriched in low-score group. High-HIF-1 score inversely correlated with CD8+ T cell density		[24]
			PDAC ICGC	High vs. Medium vs. Low	OS	2.436 × 10^−05^	NA	NA				
			GSE79668	High-risk vs. Low-risk	OS	1.246 × 10^−04^	NA	NA				
4 (*ENO3*, *LDHA*, *PGK1*, *PGM1*)	Network analysis of protein interactions of 200 genes of hallmark HYPOXIA gene-set—50 DEGs with highest interaction- 4 DEGs maintained association with survival following multivariate Cox regression analysis	Multiplying expression of nine genes with their corresponding multivariable Cox regression coefficient—classification into high- and low-hypoxia risk score based on the median risk score	PAAD TCGA	High-risk (88) vs. Low-risk (89)	OS	<0.001	1.986 (1.579–2.498) *p* < 0.001	1.878 (1.498–2.354) *p* < 0.001	CIBERSORT Expression of genes unfavorably regulating immune-related processes. Expression of genes positively regulating T cells, DCs and MDSCs	Resting NK cells Higher expression of *VEGFA*, *MICB* and *ICAM1.* Higher expression of CXCL5	CD8+ T cells, and naive B cells Higher expression of CCL21 and CCR7	[142]
			GSE78229 and GSE57495	High-risk (58) vs. Low-risk (54)	OS	0.024	1.410 (1.190–1.670) *p* < 0.001	1.622 (1.050–2.507) *p* = 0.029				
8 (*ANKZF1*, *CITED*, *ENO3*, *JMJD6*, *LDHA*, *NDST1*, *SIAH2*, *TES*)	Correlation between 200 genes of hallmark HYPOXIA gene-set and RNA-seq data of PAAD TCGA cohort—108 DEGs were correlated—45 DEGs were associated with OS based on univariate Cox regression analysis—eight maintained association based on LASSO regression analysis	Multiplying expression of eight genes with their corresponding LASSO coefficient—classification into high- and low-hypoxia risk score based on the median risk score	PAAD TCGA	High-risk (81) vs. Low-risk (81)	OS	<0.0001	2.508 (1.575–3.992) *p* < 0.0001	2.503 (1.483–4.226) *p* < 0.0001	CIBERSORT (applied only in TCGA cohort) Expression of immune checkpoint genes (applied only in TCGA cohort)	Neutrophils with higher expression of *CD47*	Treg higher expression of *BTLA*, *CTLA4*, *LAG3*, *TNFRSF4* and *PDCD1*	[143]
			GSE62452	High-risk (33) vs. Low-risk (32)	OS	0.00075	NA	NA				
3 (*ANXA2*, *LDHA*, *TES*)	Overlap between 200 genes of hallmark HYPOXIA gene-set and RNA seq data of PAAD TCGA cohort—67 DEGs correlated with OS based on univariate Cox regression analysis—three maintained association with survival following multivariate Cox regression analysis	Multiplying expression of three genes with their corresponding multivariable Cox regression coefficient—classification into high- and low-hypoxia risk score based on the median risk score	PAAD TCGA	High-risk vs. Low-risk	OS	0.00061	2.5746 (1.6083–4.122) *p* < 0.001	NA	CIBERSORT	M0 macrophages, monocytes (ICGC and GSE57495)	CD8+ T cells (TCGA and ICGC), naïve B cells (TCGA and GSE57495)	[144]
			PDAC ICGC	High-risk vs. Low-risk	OS	0.004	3.0760 (1.7135–5.522) *p* < 0.001	NA				
			GSE57495	High-risk vs. Low-risk	OS	0.031	NA	NA				

^a^ Univariate and multivariate Cox PH analysis reporting the hazard ratio, in bold, with the 95% confidence interval in brackets and corresponding *p* value. ^b^ Reported immune cell fractions present in at least two datasets. ^c^ Some studies have not reported the exact patient number per group. ^d^ Gene list was not reported. KM: Kaplan–Meier; PH: proportional hazard; Ref: reference; DEGs: differentially expressed genes; OS: overall survival; PFS: progression-free survival; DSS: disease-specific survival; DFS: disease-free survival; vs.: versus; NA: not available; Treg: regulatory T cells; TIL: tumor-infiltrating lymphocytes; DCs: dendritic cells; MDSCs: myeloid-derived suppressor cells.

The ideal hypoxia biomarker must reflect tumor oxygenation, be non-invasive and non-toxic, simple to perform, should enable repeated measures in longitudinal studies, and importantly be predictive of outcome. In that respect, the translatability of hypoxia gene signatures to the clinic is limited by the need for their prospective validation, verification of their representation of oxygen levels, defining cutoffs to indicate the degree of hypoxia, tissue requirement and the potential impact of tumor heterogeneity. Despite such constraints, the multiparametric nature of hypoxia signatures have shown them to be refractory to intra-tumoral heterogeneity, at least in HNSCCs and cervical cancers (reviewed in [29]). Moreover, several hypoxia signatures have been found to be predictive of responses to hypoxia-modifying therapy (reviewed in [29]), making them an exciting frontier to prospectively validate and translate to the clinic.

## 7. Conclusions and Future Directions

**Figure 2:**
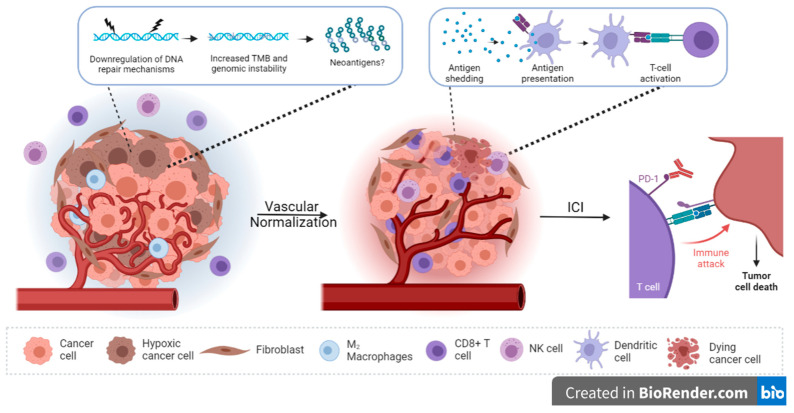
Vascular normalization to restore the anti-tumor immune response in PDAC. Vascular normalization is expected to reverse the immunosuppressive tumor microenvironment, thus enabling response to immune checkpoint inhibitors (ICIs). In addition, the effect of hypoxia on increased tumor mutational burden (TMB) and genomic instability could potentially increase the production of neoantigens. The uptake of such foreign antigens and their presentation to T cells could then enable tumor-cell killing.

Hypoxia in PDAC is associated with immunosuppression and tumor resistance and plasticity; however, accumulating evidence indicates that hypoxic stress promotes genomic instability, which could lead to improved immunogenicity of tumor cells. Given the increased interest in neoantigens, and neoantigen quality in this disease, how they are being modulated by hypoxia deserves further attention. This is of vital importance since hypoxia is not a homogenous feature in the TME and both inter- and intra-tumoral heterogeneity have been reported in PDAC. Considering the key role that hypoxia plays in PDAC pathogenesis, alleviating this condition may presumably have great potential in enhancing patient outcomes. One all-encompassing approach is through the normalization of the vasculature itself, which promotes reoxygenation of the tumor mass. ITPP is a prime candidate for successfully translating this approach to the clinic, and more work is required to determine whether this non-toxic agent can additionally reinvigorate the immune response, cumulatively enhancing the efficacy of immune checkpoint inhibitors (Figure 2). It would also be interesting to investigate whether the inherent hypoxic levels of tumors treated with ITPP, as well as other hypoxia-targeted strategies, could impact treatment effectiveness and whether hypoxia itself could act as a predictive marker of response. For that to be achieved, at least one hypoxia-detection method needs to be prospectively validated in a large multicentered clinical trial.

## Figures and Tables

**Table 1:** Pharmaceutical targeting of hypoxia in PDAC.

Target	Compound	Reference
Hypoxia	Inositol-trispyrophosphate (ITPP)	NCT02528526 [81,84]
Hypoxia-inducible factors (HIF)	XL888	NCT03095781
	Tanespimycin	NCT00577889 [85]
	AUY922	NCT01484860 [86]
	Acriflavine	[82]
Hypoxia-activated prodrugs (HAP)	Evofosfamide	NCT02402062 [87], NCT00743379, NCT02047500 [83], NCT01381822, NCT03098160 [88]
	Mitomycin	[89]
	Tirapazamine	[90]
	Apaziquone	[90]
Metabolism	BPM31510	NCT02650804
	NIR178	NCT03207867
	CPI444	NCT03454451
	Zoledronic acid	NCT00892242
	Epacadostat	NCT03006302
Immunity	BMS813160	NCT03184870, NCT03767582
	BL8040	NCT02907099, NCT02826486
	Olaptesed	NCT03168139
	Plerixafor	NCT03168139
Chemokines/cytokines	Galunisertib	NCT02734160 [91]
	Vactosertib	NCT02154646
	AP21009	NCT00844064
	M7824 fusion protein	[92]
	NIS793 mAB	[28]
	Tocilizumab	NCT02767557
	Siltuximab	NCT00841191

**Table 3:** Application of PET radiotracers for the detection of hypoxia in patients with PDAC.

PET Radiotracer	Patients with Pancreatic Cancer	Hypoxia-Related Parameter(s)	Other Parameters	Hypoxic Fraction	Other Associations	Ref.
^18^F-FMISO	Seven patients with PDAC	SUVmax and TBR (considering background uptake in skeletal muscle)	^18^F-FDG PET/CT imaging to demarcate the tumor zone. CT or MRI in non-distinguishable tumors to identify a suitable ROI	28% increased uptake values	- No association between ^18^F-FMISO SUVmax or TBR with tumor size, histological type or metabolic activity.	[134]
	25 patients with PDAC	Peak tumor–blood ratio: SUVpeak of ^18^F-FMISO in the tumor divided by SUVpeak of the aorta	Tumor ROIs were manually registered by two nuclear medicine physicians IHC of HIF-1α in 22 patients	36% visually positive for ^18^F-FMISO	- Patients with high peak tumor–blood ratio experienced worse RFS and OS. - Ratio not associated with HIF-1α.	[135]
^18^F-HX4	13 patients with PDAC	TBRmax: SUVmax in the VOI divided by average SUV in the aorta. VOI and HV: all voxels in the tumor VOI with a TBR > 1	-	High repeatability of the amount and location of elevated ^18^F-HX4 uptake	- TBRmax values were more stable compared to the SUVmax and they varied by 16%.	[136]
^18^F-FAZA	20 patients with locally advanced or metastatic PDAC (four cases with both primary tumor and liver metastasis were evaluated)	HF: percentage of voxels with SUVs more than three standard deviations from the mean SUV of skeletal muscle, as obtained from two-hour static scans	Tumor perfusion: based on tracer kinetics by acquiring dynamic scans minutes after the injection of ^18^F-FAZA and applying a two-compartment model, including blood and extravascular space, under the assumption that pancreatic tumors have low perfusion (flow limited).	Heterogeneity in the HF ranged from values less than 5% to those greater than 50%	- No correlation with tumor volume or perfusion. - Similar ^18^F-FAZA SUV reported in primary and metastatic tumors. - A trend of higher HF in primary tumors in patients with metastasis than those who are metastasis-free.	[137]

FMISO: fluoromisonidazole; FAZA: fluoroazomycin–arabinofuranoside; HX4: flortanidazole; SUVmax: maximum standardized uptake value; TBR: tumor-to-background ratio; ROI: region of interest; VOI: volume of interest; HV: hypoxic volume; HF: hypoxic fraction.
